# Physical and Mechanical Properties of Cement Mortars with Recycled Polyethylene Terephthalate: Influence of Grain Size and Composition

**DOI:** 10.3390/ma18061378

**Published:** 2025-03-20

**Authors:** Andrea Petrella, Francesco Todaro, Pravendra Yadav, Jennifer Gubitosa, Michele Notarnicola

**Affiliations:** 1 Dipartimento di Ingegneria Civile, Ambientale, Edile, del Territorio e di Chimica, Politecnico di Bari, Via E. Orabona, 4, 70125 Bari, Italy; francesco.todaro@poliba.it (F.T.); p.yadav@phd.poliba.it (P.Y.); michele.notarnicola@poliba.it (M.N.); 2 Dipartimento di Chimica, Università di Bari “Aldo Moro”, Via E. Orabona, 4, 70125 Bari, Italy; jennifer.gubitosa@uniba.it

**Keywords:** polyethylene terephthalate, cement mortars, thermal insulation, mechanical strength, wettability

## Abstract

Polyethylene terephthalate (PET) with different grain size after grinding (fine and coarse) was recycled and used as aggregate for non-conventional lightweight cement mortars. The physical and mechanical characteristics were compared to conventional sand-based composites. The workability in the fresh state was evaluated. Accordingly, the composites showed decreases in fluidity with increases in PET percentage weight. Higher thermal insulation and lower mechanical strengths were observed with the increase in plastic dosage due to a density decrease and porosity increase in the composites. Finer grain size PET samples were more resistant (~12–24 MPa) than the coarse-grain samples (~3–23 MPa) due to the higher density and specific surface area of the aggregate. Conversely, higher thermal insulation was obtained with coarse PET addition (~0.6–0.2 W/mK vs. ~0.7–0.35 W/mK). A ductile behavior with discrete cracks after failure was observed after plastic addition to the mixture. Low wettability was observed in PET samples which, although more porous than the sand specimens, showed a hydrophobic behavior which contributed to water repellency. The reported physical, mechanical, thermal, wettability and microstructural features suggest the potential of these composites for both inside and outside applications of non-structural objects.

## 1. Introduction

In the last decades, the production of plastic materials has grown dramatically and million tons of plastics are being produced worldwide for use in packaging, electronics, agriculture, building and construction applications, textiles, household and sports equipment, industrial machinery and vehicles [1,2]. Accordingly, plastic pollution has become one of the most pressing environmental issues because the massive use of this material and its inadequate management have led to an unprecedented accumulation of waste around the world [3,4,5,6].

Plastics often contain additives that contribute to improved mechanical strengths, flexibility and durability. Many of these substances, however, can increase the life of the products to hundreds of years before degrading. As a result of this biopersistence, the accumulation of plastic materials in the environment (oceans, land and urban areas) has led to devastating impacts on ecosystems and human health [7,8]. Seas and oceans are invaded by plastic with the so-called plastic islands floating in the Pacific, in the Atlantic and in the Indian Ocean [9].

Polyethylene (PE), polypropylene (PP), polyethylene terephthalate (PET), polystyrene (PS), polyvinyl chloride (PVC), polymethyl methacrylate (PMMA), polycarbonate (PC), polytetrafluoroethylene (PTFE), acrylonitrile butadiene styrene (ABS) and polyamide (PA) are the most used plastic materials.

Polyethylene terephthalate, also known as PET, is a thermoplastic polymer that belongs to the polyesters.

PET has excellent chemical resistance and barrier properties, lightness, rigidity, wear and abrasion resistance and cheapness [10,11]. These features have contributed to increases in its application in the package industry since the late 1970s. It is very stable at room temperature and decomposes at 450 °C [12,13]. Accordingly, with an estimated average life of around 1000 years, this material can be considered non-biodegradable. Fully recyclable, it does not lose its fundamental properties during the recovery process and can, therefore, be transformed repeatedly to make new products [14,15,16,17].

Reducing packaging and its correct disposal by consumers can be considered the most effective ways to reduce the environmental impact of PET. In contrast to disposal by incineration or landfilling, the conversion of this waste into a new resource through recycling operations is the most sustainable form of management because it allows for environmental protection, saving of resources and raw materials, and reducing CO_2_ emissions, while it also has economic advantages [18,19].

The use of recycled PET products in construction materials as mortars or concrete is an economical and sustainable solution that reduces the use of raw materials, mitigates the overuse of landfills and increases environmental protection. Moreover, the recycling operation in cement conglomerates improves energy savings because of the low density and high thermal insulating properties of the final products [20,21,22].

In this paper, recycled polyethylene terephthalate (PET), derived from bottle scraps, was used as aggregate in eco-innovative cement mortars, with the aim to reduce the plastic’s environmental impact and raw material consumption.

The main properties of cement conglomerates with PET have been investigated. It was observed that its use as a replacement for conventional aggregate resulted in decreases in mechanical strength but also a reduction in density and water absorption [23,24,25,26], together with increases in deformational properties such as expansion or contraction and freezing and thawing under harsh weather conditions [27].

It was also observed that the thermal conductivity of the composite decreased with the increase in plastic waste; accordingly, PET composites slowed down thermal heat transfer with respect to conventional construction materials [28,29,30]. Chemical treatments have also been carried out to improve the surface properties of plastic aggregates. For this purpose, depolymerized PET (DPET) aggregates were used in the production of construction materials to improve the mechanical properties of these composites [31,32,33]. The most common methods of PET waste depolymerization under various reaction conditions are based on water (hydrolysis), glycols (glycolysis), amines (aminolysis), and alcohols (alcoholysis) [32].

In this research, we investigated recycled PET of different grain sizes after grinding (fine and coarse) and without other pretreatments before use. Moreover, the mixture preparation did not require additives or expensive procedures. A partial and complete substitution of sand was carried out. The workability, thermo-mechanical properties, wettability and microstructural characteristics were analysed through evaluation of the grain size effect and comparison with conventional conglomerates. The aim was to identify the best performing lightweight thermal-insulating mortars for non-structural applications in buildings, for inside and possibly outside elements, as plasters and for vertical elements in general.

## 2. Materials and Methods

### Preparation of the Mortars and Methods of Characterization

The aim of the research was to investigate the effects of the addition of recycled untreated PET of different sizes on the workability, thermo-mechanical, microstructural and wettability properties of cement mortars. For this purpose, the samples were prepared with increasing %wt of PET as a sand replacement in order to understand the influence of gradual reductions in the conventional aggregate and its replacement with lightweight recycled material. Moreover, the effect of different PET grain sizes was analysed by the use of two types, namely PETf (fine) and PETc (coarse), whose geometry can affect the mortar’s properties.

The cement mortars were prepared with CEM II A-LL 42.5 R (Buzzi Unicem, Barletta, Italy), which is composed of 80–94% clinker, 6–20% limestone LL (<0.2% organic carbon), gypsum (0–5%), and minor additional constituents, with a Blaine specific surface area in the range of 3100–4400 cm^2^/g, and Rc (28 days) > 47.0 MPa [34].

**Figure 1:**
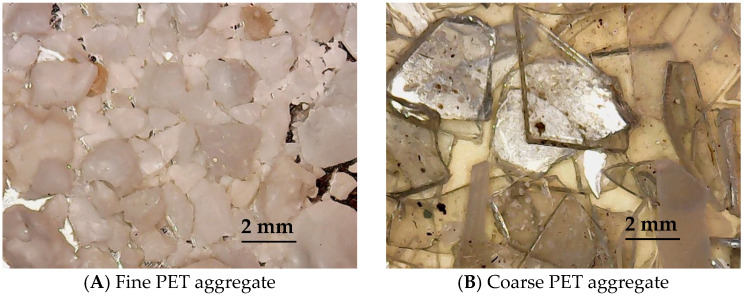
Fine PET and coarse PET aggregates used for mortar preparation.

Polyethylene terephthalate (PET) from bottle scraps [15] (Maltek Industrie Srl, Terlizzi, Italy) was crushed (with the cutting mill SM 300, Retsch GmbH, Haan, Germany) and used after sieving (with electromagnetic sieve shaker IRIS FTL-0200, FILTRA Sl, Barcelona, Spain). The PET grain size was in the range of 1–2 mm (PETf) and 2.5–3.5 mm (PETc), and the PET was used to partially and totally substitute sand aggregate (Figure 1).

Normalized sand (~1700 g/dm^3^, 0.08–2 mm) from Societè Nouvelle du Littoral (Leucate, France) was used as received and had a grain size in the range of 0.5–1 mm.

The PET and sand were used as aggregates in conglomerates prepared with 225 g of water and 450 g of cement [35], with increasing percentage weights of PETf and PETc as a sand replacement.

A normalized mortar (450 g cement, 225 g water and 1350 g normalized sand) and two sand specimens (S1 and S2) containing 500 g and 250 g, respectively, of 0.5–1 mm sand were prepared as references. The choice of the two other references was based on the PET weight to be added for the mortars’ preparation (250 g and 500 g).

The samples with partial sand replacement were named as SPf and SPc, while samples with bare PET were denoted as PET samples. Accordingly, SPf1, SPf2, SPf3, SPf4, PET1f and PET2f were conglomerates prepared with fine PET aggregate, while SPc1, SPc2, SPc3, SPc4, PET1c and PET2c were conglomerates prepared with coarse PET aggregate. Finally, a mixture of both PET aggregates was denoted as PETf/PETc.

Table 1 reports the composition of the specimens.

The consistency of the mixtures in the fresh state was determined by a flow-test [36]. In this respect, the percentage increase in the mortar’s diameter over the base diameter was evaluated with the following equation: flow = [(D2 − D1)/D1] × 100, where D2 is the average spread diameter and D1 is the original base diameter. The Δflow was obtained starting from the normalized mortar spread diameter (18 cm) and evaluating the spread diameter of each mixture as percentage variation (increase or decrease). The aim was to understand the behavior of the novel mortars in the fresh state with respect to the conventional sand conglomerate.

The mixtures in the hardened state were characterized by mechanical and thermal tests. For the mechanical tests, 40 × 40 × 160 mm prisms were prepared and cured for 7, 28 and 60 days [37,38]. Afterwards, flexural (50 ± 10 N/s loading rate) and compressive (2400 ± 200 N/s loading rate) loads were applied (MATEST device, Milan, Italy). For the thermal measurement tests, *ϕ* = 100 mm; h = 50 mm cylinders were cured for 28 days then thermally characterized by an ISOMET 2104 device (Applied Precision Ltd., Bratislava, Slovakia). A heating probe placed on the front face of the sample produced a constant thermal flow which provided an estimation of the thermal conductivity (*λ*) of the specimens [39].

The microstructural characterization of the samples was carried out by a FESEM-EDX Carl Zeiss Sigma 300 VP (Carl Zeiss Microscopy GmbH, Jena, Germany) after gold sputtering (Sputter Quorum Q150 Quorum Technologies Ltd., East Sussex, UK). The aggregate nature and distribution in the specimens were observed with a Premier series dyno-lyte portable microscope with background cold lighting, which enabled observation of the wettability tests.

Porosimetric measurements were obtained with an Ultrapyc 1200e Automatic Gas Pycnometer (Quantachrome Instruments, Boynton Beach, FL, USA), using helium gas.

## 3. Results

### 3.1. Cement Mortar Composition

Figure 1 shows an image of both types of PET after grinding, before the introduction as aggregates into the cement mixtures.

Table 1 reports the composition and physical properties of the conglomerates. The addition of a higher percentage weight of aggregate (500 g, S1) resulted in an increase in density and a slight decrease in porosity, in the case of sand samples with respect to the addition of 250 g of aggregate (S2). Conversely, the samples PET1f and PET1c with 500 g of aggregate, were lighter and had higher porosity than PET2f and PET2c with 250 g of aggregate because of the higher dosage of lightweight plastics. The bare sand samples (Ref, S1 and S2) showed the highest density and the lowest porosity, whereas the addition of PET (SP specimens) led to increases in lightness and porosity, which reached the highest values in the samples with total sand replacement (PET1f, PET2f, PET1c, PET2c, PETf/PETc). The conglomerates with coarse PET were lighter than the conglomerates with fine PET due to the lower density of the former aggregate.

**Figure 2:**
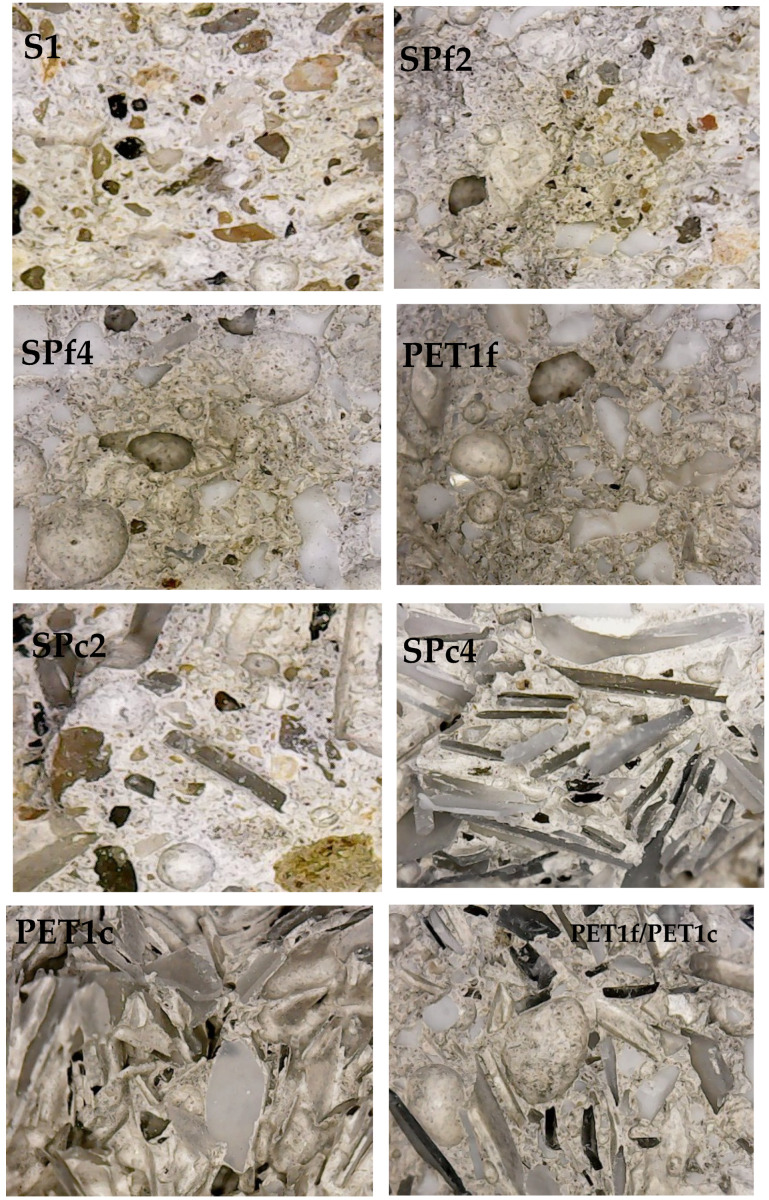
Sections of the cement mortars.

The different natures, compositions and distributions of the aggregates can be observed in Figure 2, where the sections of the specimens are shown. It can be observed that there is a good aggregate distribution in the mortars, and there are increases in PET percentage weight from S1 specimen (without PET) to the PET1f and PET1c specimens (with total sand replacement). Moreover, the different grain sizes of the organic material are evident, also in the specimen with 50% PETf and 50% PETc (PETf/PETc).

### 3.2. Consistency of the Mortars in the Fresh State

**Figure 3:**
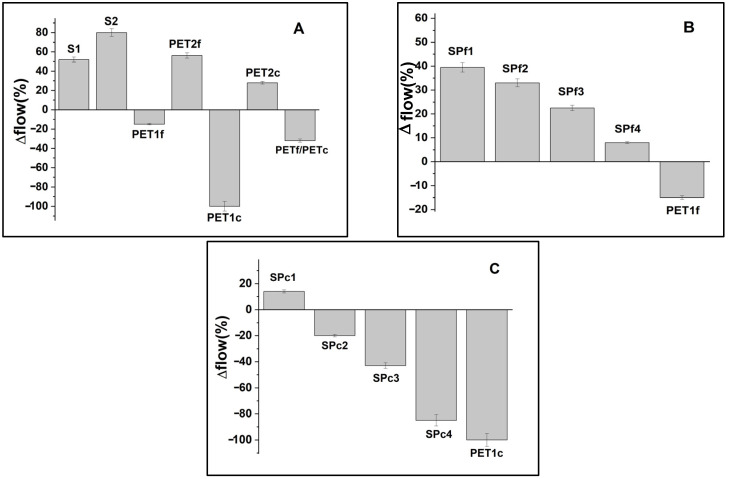
Flow variations with respect to the normalized mortar of (**A**) bare sand and PET composites, (**B**) sand/fine PET, and (**C**) sand/coarse PET composites, as a function of waste addition.

The consistency of the fresh specimens was determined by flow-test measurements (Figure 3), and the results were compared to the control (∆ flow) [36]. Figure 3A shows flow variations with respect to the normalized mortar of bare sand and PET composites, while Figure 3B,C show the flow variation for the sand/fine PET composites (SPf samples) and sand/coarse PET composites (SPc samples), respectively. S1 and S2 (Figure 3A) contained more fluid than the normalized mortar (+50% and +80%) due to the lower %wt of sand (500 g and 250 g, respectively) and to the lack of fines. The PET samples contained less fluid than the sand samples due to the higher volume of the organic aggregate with respect to sand. PET1f showed similar workability to that of the reference (−20%) and contained more fluid than PET1c (−100%) due to the lower volume at the same dosage, which increased the fluidity, while PETf/PETc showed an intermediate trend. PET2f and PET2c, with lower PET %wt (250 g), were more fluid than the samples with higher PET %wt (PET1f and PET1c, with 500 g of aggregate).

The SP composites showed decreases In fluidity with Increases In PET %wt as sand replacement (Figure 3B,C), with SPf4 (+10%) and SPc1 (−10%) showing behavior similar to the normalized mortar. The lower fluidity attributed to the addition of coarse PET is confirmed in the SPc specimens with respect to the SPf composites.

### 3.3. Thermal Measurements

**Figure 4:**
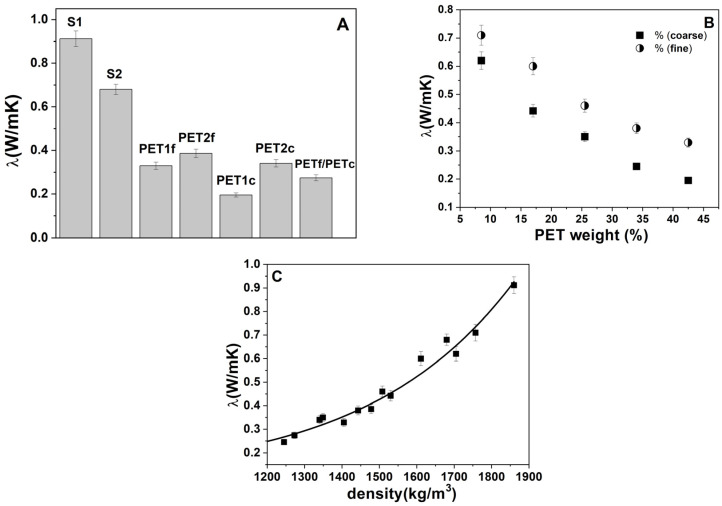
Thermal conductivity of (**A**) bare sand and PET composites; (**B**) sand/fine PET and sand/coarse PET composites as a function of waste addition. (**C**) Thermal conductivity as a function of the composite density. Normalized mortar thermal conductivity = 2.0 W/mK.

**Figure 5:**
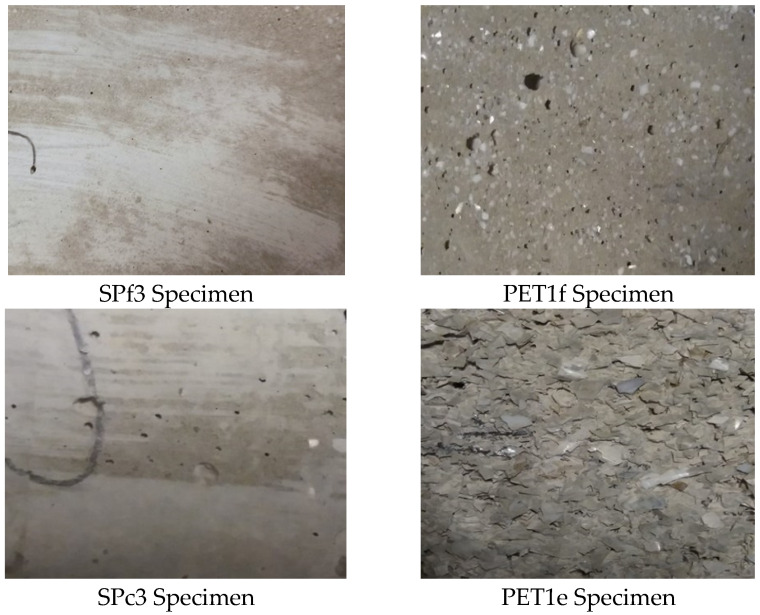
External surface of SPf3, PET1f, SPc3 and PET1c specimens.

Figure 4A shows the thermal conductivity of the bare sand and PET composites. The S1 and S2 specimens showed lower thermal conductivity (~0.7–0.9 W/mK) than the normalized mortar (~2 W/mK), attributed to a lower content of aggregate (500 g and 250 g vs. 1350 g) and a lack of fines. Moreover, the bare PET samples were more insulating than the sand specimens, which was associated with the addition of the low-density aggregates [37,40]. The PETc composites showed lower thermal conductivity than the PETf composites due to the higher volume, with a constant dosage. Figure 4B shows results for the sand/fine PET and sand/coarse PET composites as a function of plastic material addition. An increase in the thermal insulation of the SP samples was obtained with the introduction of PET waste into the mixture, with values in the range of ~0.6–0.2 W/mK, in the case of PETc composites, and values in the range of ~0.7–0.35 for the PETf composites. Accordingly, the mortars with the best performances were PET1f and PET1c with total sand replacement. These results are ascribed to a decrease in density and increase in porosity with the *w*/*w*% of organic added. In fact, Figure 4C shows the thermal conductivity of the composites as a function of density, where an exponential increase in λ was observed with the increase in the density of composites [41,42,43]. Moreover, the increase in porosity in the samples with total sand replacement (PET1f and PET1c) can be observed in Figure 5, where the external surfaces of the PET samples and SP specimens are shown. In the case of the SP mortars, a smooth surface is observed.

### 3.4. Mechanical Tests

**Figure 6:**
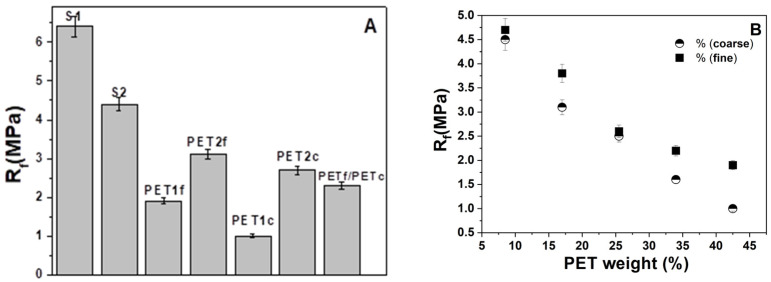
Flexural strength results of (**A**) bare sand and PET composites, and (**B**) sand/fine PET (SPf) and sand/coarse PET (SPc) composites as a function of waste addition. Normalized mortar flexural strength = 8 MPa.

Figure 6A reports the flexural strengths results of bare sand and PET composites, the former with higher resistances (~4.5 MPa–6.5 MPa vs. ~1–3.5 MPa) due to the presence of inorganic material. As expected, the S1 and S2 results were lower than the normalized mortar (~8 MPa) due to the different aggregate composition and dosage. The presence of finer-grain PET resulted in increased strength with respect to coarse-grain PET because of the higher density of the samples, while intermediate values were observed with the mixture of organic aggregates (PETf/PETc).

Figure 6B reports flexural strength results for the sand/fine PET (SPf) and sand/coarse PET (SPc) composites as a function of waste addition. The higher strengths of SPf samples were confirmed, and decreases with the plastic %wt were observed for both types of mortars. The explanation for this result is based on the decrease in density together with the increase in porosity [37].

**Figure 7:**
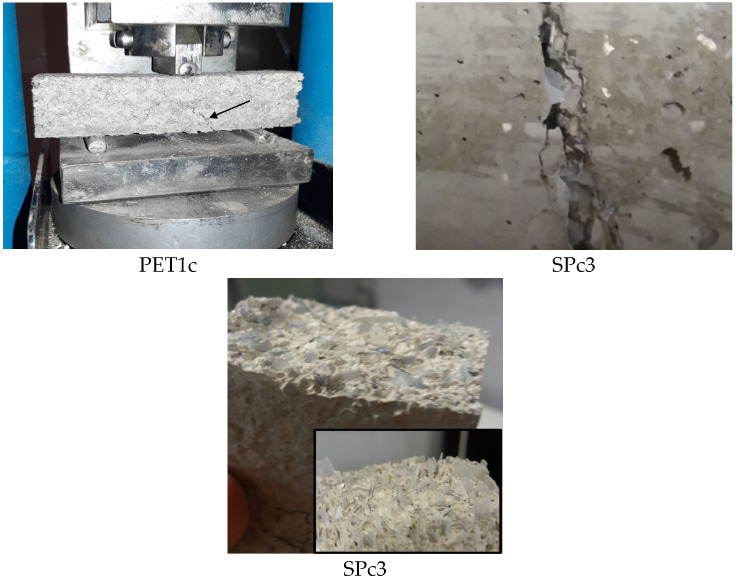
Failure of PET1c and SPc3 composites together with SPC3 breaking surface.

The failure mode was brittle for the sand samples, semi-brittle for the SP1 and SP2 samples, and finally, ductile for the SP3, SP4 and PET samples. Figure 7 shows the failure of the PET1c and SPc3 composites, characterized by the presence of discrete cracks after failure without collapse, which is associated with the flexibility and energy absorption capacity of the plastic material. Moreover, the SPC3 breaking surface shows the PET aggregates orientation in the conglomerates which prevents the propagation of cracks.

**Figure 8:**
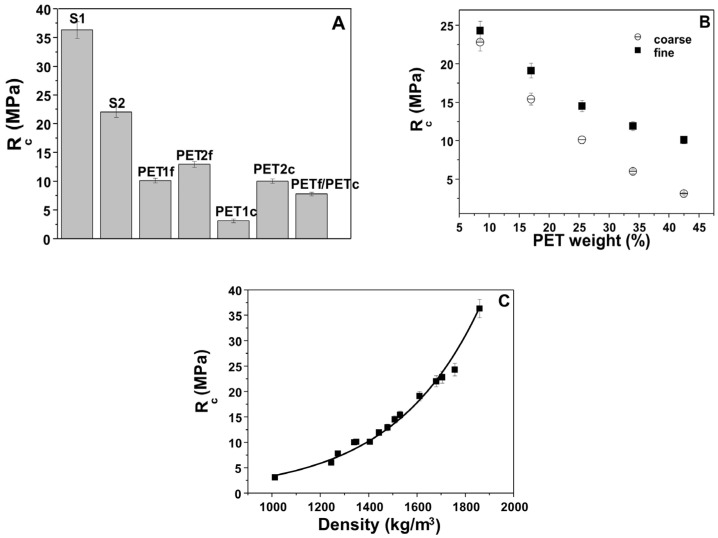
Compressive strengths of (**A**) bare sand and PET composites, and (**B**) sand/fine PET and sand/coarse PET composites as a function of waste addition. (**C**) Compressive strengths as a function of the composite density. Normalized mortar compressive strength = 50 MPa.

Figure 8A reports the compressive strengths results of the bare sand and PET composites. Also in this case, as in the flexural resistance observations, the sand samples (S1 and S2) were more resistant (~22–36 MPa) than the other specimens with plastic (~4–13 MPa). Moreover, the S1 and S2 strengths were lower than that of the normalized mortar (~50 MPa) due to the different aggregate composition and dosage.

Figure 8B reports the sand/fine PET (SPf) and sand/coarse PET (SPc) composites as a function of waste addition. The increase in percentage weight of plastic led to decreased mechanical strength, which was associated with increased porosity [37,38]. Moreover, the resistances of the PETf composites were higher than those of the PETc composites, and the difference between the two types of PET was higher with the increase in plastic addition. This result could be ascribed to the larger specific surface of the finer aggregates which induced increases in compaction and density.

Finally, an exponential increase in compressive strength was observed with increases in the density of the composites (Figure 8C).

**Figure 9:**
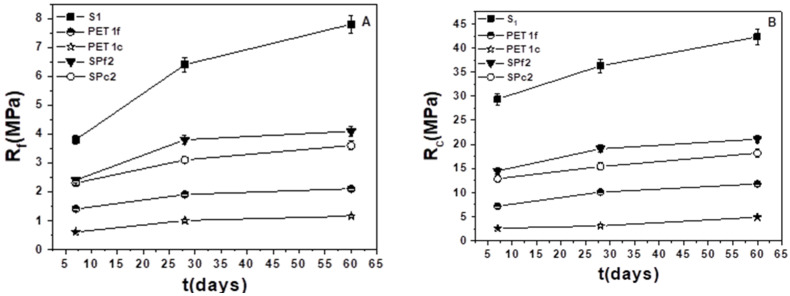
(**A**) Flexural and (**B**) compressive strengths over time of the sand, PET, and sand/PET samples.

The stability of the flexural and compressive strengths over time can be observed in Figure 9A,B, with a slight increase over the longer period, which confirms that the mix design of the mortars is appropriate.

### 3.5. SEM Characterization of the Samples

An explanation of the thermal and mechanical results can be found through the SEM analysis, which shows that the geometry and uniformity in the shape of the PET aggregate can affect its adhesion to the cement paste.

**Figure 10:**
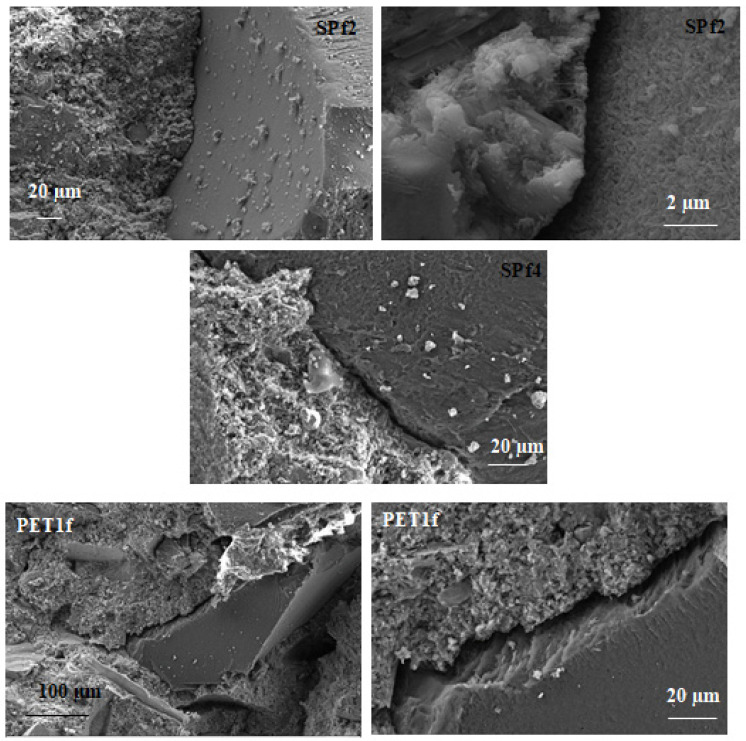
Scanning electron microscopy images for SPf2 (~1.5 µm voids at the interface), SPf4 (~3 µm voids at the interface) and PET1f (~6 µm voids at the interface).

Figure 10 shows scanning electron microscopy observations for the SPf2, SPf4 and PET1f samples. It is evident that there is a lack of adhesion of the organic aggregate to the cement paste because of the different chemical nature of the conglomerate components, characterized by hydrophobic (PET) and hydrophilic (cement) domains. This is in contrast to the strong bonding observed between the cement matrix and the natural sand. As expected, the interface separation shows an enlargement with PET addition; in fact, SPf2 showed ~1.5 µm voids at the interface, SPf4 had ~3 µm voids at the interface, and PET1f ~6 µm voids. This result is ascribed to the lower cement/aggregate ratio. This enlargement with increasing amounts of PET in the mixtures caused difficulty in the workability and resulted in a more porous structure of the final products. This can also explain the decrease in the mechanical strengths that is also associated with lower PET stiffness, and the increase in thermal insulation with respect to the standard sand specimens.

**Figure 11:**
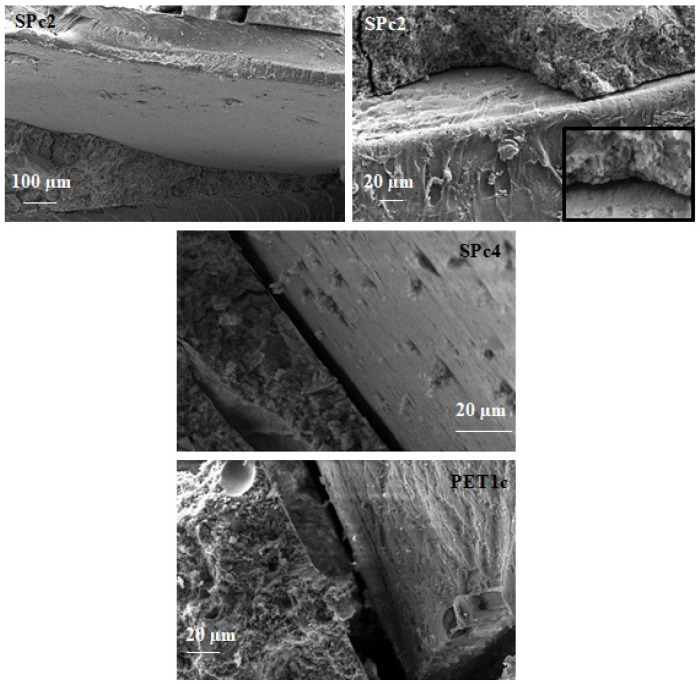
Scanning electron microscopy images for SPc2 (~2.5 µm voids at the interface), SPc4 (~7 µm voids at the interface) and PET1c (~20 µm voids at the interface).

Similar behavior was observed in Figure 11, where the introduction of coarse PET led to weaker bonding of the organic/inorganic domains, with ever increasing separation at increasing %wt. In fact, the SPc2 sample showed ~2.5 µm voids, SPc4 had ~7 µm voids, and PET1c ~20 µm voids at the interface. The reduction in workability was even more evident for these mixtures, as was the increase in porosity (density decrease), which led to reductions in resistance and thermal conductivity with respect to the PETf mortar samples.

The fine PET grains show a higher specific surface area, which can result in better adhesion to the cement paste, with respect to the coarse PET aggregate, which has rougher and more well-defined edges than the fine version and lower specific surface area. Fine aggregate PET promotes pores with smaller sizes, whereas coarse aggregate PET promotes larger pores.

### 3.6. Wettability Tests

**Figure 12:**
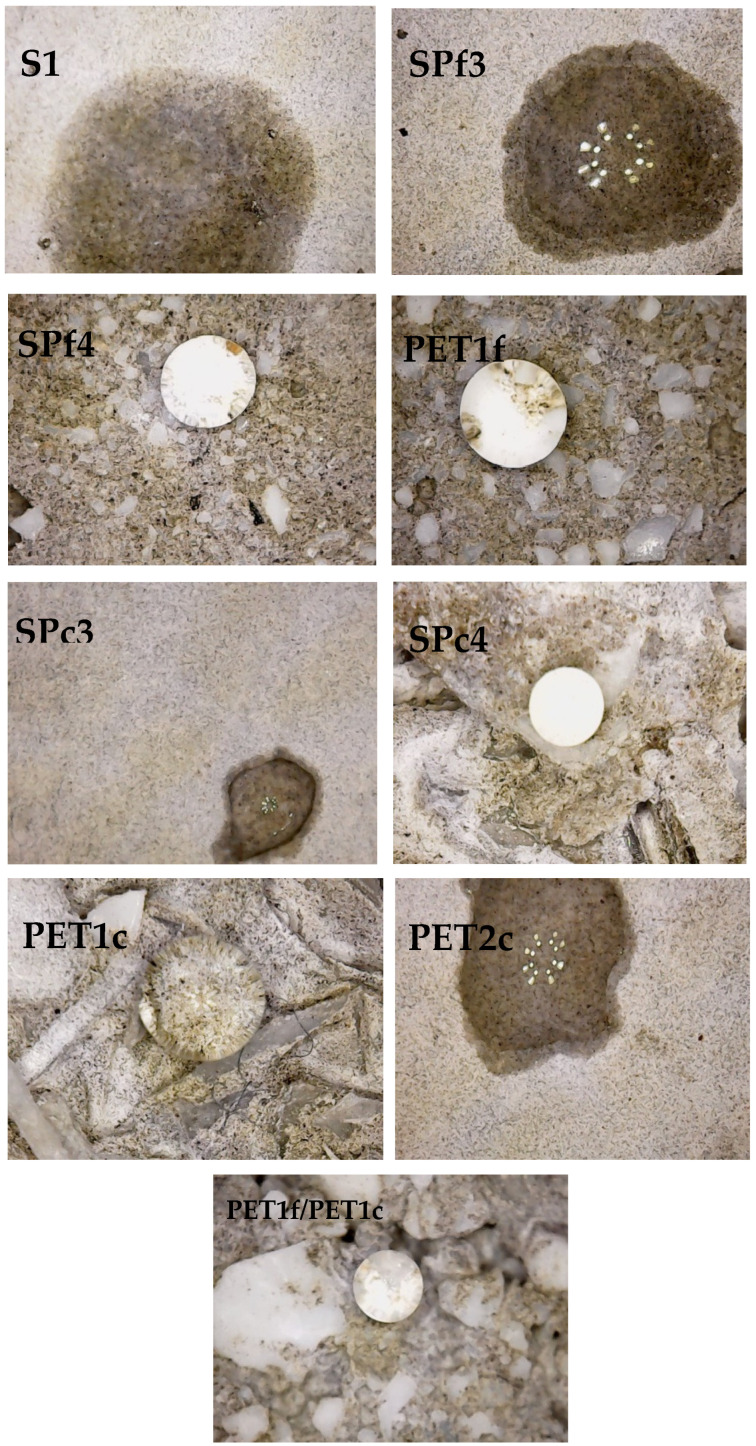
Wettability tests of the specimens on the external surface.

Figure 12 shows the wettability tests of the specimens. Specifically, poor wettability is ascribed to hydrophobic behavior of a surface (water contact angle > 90°); conversely, good wettability is ascribed to hydrophilic behavior of a surface (water contact angle < 90°) [44].

For the case at hand, sand specimens (Ref, S1, S2), characterized by hydrophilic features, together with cement phase porosity, showed fast water absorption. The PET samples, although more porous than the sand samples (as observed in microscopical images), were characterized by hydrophobic behavior because of the hydrophobic features of the plastic aggregate [37]. This is evident in the PET1f, PET1c and PETf/PETc observations of Figure 12. The sand/PET specimens showed wettability in the case of SP1 and SP2, low wettability in the case of SP3, and hydrophobic behavior for the SP4 composites. Therefore, the contribution of the hydrophobic domains of plastic tends to be predominant in the samples with 300 g of PET, even in the presence of the hydrophilic porous cement paste. This effect is more evident with fine PET mortars because of the higher specific surface area of the organic aggregate. Low wettability was observed in the PET2f and PET2c composites that, in spite of the total sand replacement, contained 250 g of plastic in the mix.

### 3.7. Economic and Ecological Issues of Using PET in Cement Mortars

Compared to producing brand-new plastic, chemical recycling of PET wastes has some limitations, such as high energy consumption, high cost (e.g., 500 EUR/ton), and the need for specialized equipment. In particular, poor quality of the collected PET products (i.e., contamination of different materials) decreases the quality of the recycled raw materials and, consequently, decreases the scope for further application [45]. However, the reuse of PET scrap is beneficial if used in the production of various concrete products [46]. Indeed, utilizing PET as cement aggregate is considered an environmentally friendly method for the disposal of plastic waste [47].

The specimens tested in this work are effective and environmentally sustainable conglomerates due to their preparation with products derived from PET scraps and due to their lightness, which is fundamental for thermal insulating materials in non-structural structures. Moreover, several studies have demonstrated that the reuse of PET as aggregates in cement mortars is not harmful to health [48,49].

## 4. Conclusions

Recycled polyethylene terephthalate (PET), derived from bottle scraps, was used as an aggregate in eco-innovative cement mortars. PET with different grain sizes after grinding (fine and coarse) was recycled without pre-treatment.

The addition of PET resulted in density decreases. The mortars with coarse PET were lighter (1000–1700 kg/m^3^) than the conglomerates with fine PET (1400–1760 kg/m^3^) due to the lower density of the former aggregate.

The PET mortar samples were less fluid than the sand mortar samples due to the higher volume of the organic aggregate, and the SP composites showed a decrease in fluidity with increases in PET dosage. Fine aggregates contributed to lower workability compared to samples with coarse aggregates because of the higher specific surface area.

It was also observed that there were increases in thermal insulation with increased PET content in the mixtures. These results were ascribed to an increase in porosity. Coarse PET composites showed lower thermal conductivity than fine PET composites (~0.6–0.2 W/mK vs. ~0.7–0.35 W/mK) due to the higher volume of aggregate.

Additionally, a decrease in mechanical strengths due to the increase in porosity was observed. Finer grain size PET mortar samples showed more resistance than the coarse-grain samples (~12–24 MPa vs. ~3–23 MPa) due to the higher density and specific surface of the aggregate, and a ductile behavior with discrete cracks after failure without collapse was observed when more than 25 %wt of PET was added.

Microscopical observations showed voids at the plastic/cement interface. Moreover, increased void width with increasing %wt of PET was observed, especially in the PETc composites, which resulted in their lower density with respect to the PETf composites.

Finally, the PET mortar samples, although more porous than the sand mortar samples, were characterized by hydrophobic behavior because of the hydrophobic nature of the plastic aggregate, an effect that tends to be predominant in the samples with 25 %wt or more of PET.

Applications as non-structural and thermo-insulating components can be suggested for these lightweight composites, although further research is necessary. Specifically, the composites could find suitable applications in buildings as plasters for inside, and possibly for outside walls, and as vertical elements or panels in general.

## Figures and Tables

**Table 1:** Composition and physical properties of the conglomerates.

Type	Cement (g)	Water (cm^3^)	PETf Weight (g)	PETc Weight (g)	Sand Weight (g)	*ρ* (Kg/m^3^)	Porosity %
Ref	450	225	0	0	1350	2060	22
S1	450	225	0	0	500	1860	23
S2	450	225	0	0	250	1680	24
SPf1	450	225	100	0	400	1757	28
SPf2	450	225	200	0	300	1611	30
SPf3	450	225	300	0	200	1508	31
SPf4	450	225	400	0	100	1443	35
SPc1	450	225	0	100	400	1705	30
SPc2	450	225	0	200	300	1530	33
SPc3	450	225	0	300	200	1349	35
SPc4	450	225	0	400	100	1245	38
PET1f	450	225	500	0	0	1405	35
PET2f	450	225	250	0	0	1478	31
PET1c	450	225	0	500	0	1012	41
PET2c	450	225	0	250	0	1340	37
PETf/PETc	450	225	250	250	0	1273	36

## Data Availability

The raw data supporting the conclusions of this article will be made available by the authors on request due to privacy.

## References

[1] Bachmann M., Zibunas C., Hartmann J., Tulus V., Suh S., Guillén-Gosálbez G., Bardow A. (2023). Towards circular plastics within planetary boundaries. Nat. Sustain..

[2] Chen Y., Awasthi A.K., Wei F., Tan Q., Li J. (2021). Single-use plastics: Production, usage, disposal, and adverse impacts. Sci. Total Environ..

[3] Raheem A.B., Noor Z.Z., Hassan A., Hamid M.K.A., Samsudin S.A., Sabeen A.H. (2019). Current developments in chemical recycling of post-consumer polyethylene terephthalate wastes for new materials production: A review. J. Clean. Prod..

[4] Law K.L., Narayan R. (2022). Reducing environmental plastic pollution by designing polymer materials for managed end-of-life. Nat. Rev. Mater..

[5] Andrady A.L. (2011). Microplastics in the marine environment. Sci. Mar. Pollut. Bull..

[6] MacLeod M., Arp H.P.H., Tekman M.B., Jahnke A. (2021). The global threat from plastic pollution. Science.

[7] Thushari G., Senevirathna J. (2020). Plastic pollution in the marine environment. Heliyon.

[8] Napper I.E., Thompson R.C. (2020). Plastic debris in the marine environment: History and future challenges. Glob. Chall..

[9] Harris P.T., Maes T., Raubenheimer K., Walsh J. (2023). A marine plastic cloud—Global mass balance assessment of oceanic plastic pollution. Cont. Shelf Res..

[10] Sulyman M., Haponiuk J., Formela K. (2016). Utilization of recycled polyethylene terephthalate (PET) in engineering materials: A re-view. Int. J. Environ. Sci. Dev..

[11] Nisticò R. (2020). Polyethylene terephthalate (PET) in the packaging industry. Polym. Test..

[12] Jabarin S.A., Lofgren E.A. (1984). Thermal stability of polyethylene terephthalate. Polym. Eng. Sci..

[13] Sadeghi B., Marfavi Y., AliAkbari R., Kowsari E., Ajdari F.B., Ramakrishna S. (2021). Recent studies on recycled PET fibers: Production and applications: A review. Mater. Circ. Econ..

[14] Jang J.Y., Sadeghi K., Seo J. (2022). Chain-extending modification for value-added recycled PET: A review. Polym. Rev..

[15] Benyathiar P., Kumar P., Carpenter G., Brace J., Mishra D.K. (2022). Polyethylene terephthalate (PET) bottle-to-bottle recycling for the beverage industry: A review. Polymers.

[16] Chinchillas-Chinchillas M.J., Gaxiola A., Alvarado-Beltrán C.G., Orozco-Carmona V.M., Pellegrini-Cervantes M.J., Rodríguez-Rodríguez M., Castro-Beltrán A. (2020). A new application of recycled-PET/PAN composite nanofibers to cement–based materials. J. Clean. Prod..

[17] Gallardo-Sánchez M.A., Chinchillas-Chinchillas M.J., Gaxiola A., Alvarado-Beltrán C.G., Hurtado-Macías A., Orozco-Carmona V.M., Almaral-Sánchez J.L., Sepúlveda-Guzmán S., Castro-Beltrán A. (2022). The use of recycled PET for the synthesis of new mechanically improved PVP composite nanofibers. Polymers.

[18] Shamsuyeva M., Endres H.J. (2021). Plastics in the context of the circular economy and sustainable plastics recycling: Comprehensive review on research development, standardization and market. Compos. Part C Open Access.

[19] Oberoi I.S., Rajkumar P., Das S. (2021). Disposal and recycling of plastics. Mater. Today Proc..

[20] Mohan H.T., Jayanarayanan K., Mini K. (2021). Recent trends in utilization of plastics waste composites as construction materials. Constr. Build. Mater..

[21] Ojeda J.P. (2021). A meta-analysis on the use of plastic waste as fibers and aggregates in concrete composites. Constr. Build. Mater..

[22] Pacheco-Torgal F., Khatib J., Colangelo F., Tuladhar R. (2018). Use of Recycled Plastics in Eco-Efficient Concrete.

[23] Yilmaz A. (2021). Mechanical and durability properties of cement mortar containing waste pet aggregate and natural zeolite. Ceram. Silik..

[24] Campanhão A.F., Marvila M.T., de Azevedo A.R.G., da Silva T.R., Fediuk R., Vatin N. (2021). Recycled PET sand for cementitious mortar. Materials.

[25] Ahmad F., Jamal A., Mazher K.M., Umer W., Iqbal M. (2021). Performance evaluation of plastic concrete modified with e-waste plastic as a partial replacement of coarse aggregate. Materials.

[26] Almeshal I., Tayeh B.A., Alyousef R., Alabduljabbar H., Mohamed A.M. (2020). Eco-friendly concrete containing recycled plastic as partial replacement for sand. J. Mater. Res. Technol..

[27] Ghaly A., Gill M. (2004). Compression and Deformation Performance of Concrete Containing Postconsumer Plastics. ASCE J. Mater. Civ. Eng..

[28] Almeshal I., Tayeh B.A., Alyousef R., Alabduljabbar H., Mohamed A.M., Alaskar A. (2020). Use of recycled plastic as fine aggregate in cementitious composites: A review. Constr. Build. Mater..

[29] Akçaözoğlu S., Akçaözoğlu K., Atiş C.D. (2013). Thermal conductivity, compressive strength and ultrasonic wave velocity of cementitious composite containing waste PET lightweight aggregate (WPLA). Compos. Part B Eng..

[30] Da Luz Garcia M., Oliveira M.R., Neto-Silva T., Meira-Castro A.C. (2021). Performance of mortars with PET. J. Mater. Cycles Waste Manag..

[31] Foti D., Lerna M. (2020). New mortar mixes with chemically depolymerized waste PET aggregates. Adv. Mater. Sci. Eng..

[32] Geyer B., Lorenz G., Kandelbauer A. (2016). Recycling of poly(ethylene terephthalate)—A review focusing on chemical methods. Express Polym. Lett..

[33] Mahdi F., Abbas H., Khan A.A. (2013). Flexural, shear and bond strength of polymer concrete utilizing recycled resin obtained from post consumer PET bottles. Constr. Build. Mater..

[34] Italian Organization for Standardization (UNI) Cement Composition, Specifications and Conformity Criteria for Common Cements. EN 197-1. https://store.uni.com/uni-en-197-1-2011.

[35] Italian Organization for Standardization (UNI) Methods of Testing Cement-Part 1: Determination of Strength. EN 196-1. https://store.uni.com/uni-en-196-1-2016.

[36] Italian Organization for Standardization (UNI) Determination of Consistency of Cement Mortars Using a Flow Table. UNI 7044:1972. https://store.uni.com/uni-7044-1972.

[37] Petrella A., Di Mundo R., Notarnicola M. (2020). Recycled expanded polystyrene as lightweight aggregate for environmentally sus-tainable cement conglomerates. Materials.

[38] Petrella A., Petrella M., Boghetich G., Petruzzelli D., Calabrese D., Stefanizzi P., De Napoli D., Guastamacchia M. (2007). Recycled waste glass as aggregate for lightweight concrete. Proc. Inst. Civ. Eng. Constr. Mater..

[39] Gustafsson S.E. (1991). Transient plane source techniques for thermal conductivity and thermal diffusivity measurements of solid materials. Rev. Sci. Instrum..

[40] Petrella A., Petruzzelli V., Basile T., Petrella M., Boghetich G., Petruzzelli D. (2010). Recycled porous glass from municipal/industrial solid wastes sorting operations as a lead ion sorbent from wastewaters. React. Funct. Polym..

[41] Pásztory Z., Anh Le D.H. (2021). An overview of factors influencing thermal conductivity of building insulation materials. J. Build. Eng..

[42] Petrella A., Petrella M., Boghetich G., Basile T., Petruzzelli V., Petruzzelli D. (2011). Heavy metals retention on recycled waste glass from solid wastes sorting operations: A comparative study among different metal species. Ind. Eng. Chem. Res..

[43] Ahmed S.N., Sor N.H., Ahmed M.A., Qaidi S.M. (2022). Thermal conductivity and hardened behavior of eco-friendly concrete incorporating waste polypropylene as fine aggregate. Mater. Today Proc..

[44] Roy T., Sabharwal T., Kumar M., Ranjan P., Balasubramaniam R. (2020). Mathematical modelling of superhydrophobic surfaces for determining the correlation between water contact angle and geometrical parameters. Precis. Eng..

[45] Santomasi G., Todaro F., Petrella A., Notarnicola M., van Velzen E.U.T. (2024). Mechanical Recycling of PET Multi-Layer Post-Consumer Packaging: Effects of Impurity Content. Recycling.

[46] Joseph T.M., Azat S., Ahmadi Z., Jazani O.M., Esmaeili A., Kianfar E., Haponiuk J., Thomas S. (2024). Polyethylene terephthalate (PET) recycling: A review. Case Stud. Chem. Environ. Eng..

[47] Askar M.K., Al-Kamaki Y.S.S., Hassan A. (2023). Utilizing Polyethylene Terephthalate PET in Concrete: A Review. Polymers.

[48] Alfahdawi I.H., Osman S.A., Hamid R., Al-Hadithi A.I. (2016). Utilizing waste plastic polypropylene and polyethylene tereph-thalate as alternative aggregates to produce lightweight concrete: A review. J. Eng. Sci. Technol..

[49] Uche C.K.A., Abubakar S.A., Nnamchi S.N., Ukagwu K.J. (2023). Polyethylene terephthalate aggregates in structural lightweight concrete: A meta-analysis and review. Discov. Mater..

